# Rapid Airplane Detection in Remote Sensing Images Based on Multilayer Feature Fusion in Fully Convolutional Neural Networks

**DOI:** 10.3390/s18072335

**Published:** 2018-07-18

**Authors:** Yuelei Xu, Mingming Zhu, Peng Xin, Shuai Li, Min Qi, Shiping Ma

**Affiliations:** 1 Aeronautics Engineering College, AFEU, Xi’an 710038, China; ming_paper@163.com (M.Z.); wszxxmx@163.com (P.X.); lishuailisuai@163.com (S.L.); mashiping@126.com (S.M.); 2 Unmanned System Research Institute, Northwestern Polytechnical University, Xi’an 710072, China; 3 School of Electronics and Information, Northwestern Polytechnical University, Xi’an 710072, China; drqimin@nwpu.edu.cn

**Keywords:** remote sensing images, airplane detection, fully convolutional neural networks, feature fusion

## Abstract

To address the issues encountered when using traditional airplane detection methods, including the low accuracy rate, high false alarm rate, and low detection speed due to small object sizes in aerial remote sensing images, we propose a remote sensing image airplane detection method that uses multilayer feature fusion in fully convolutional neural networks. The shallow layer and deep layer features are fused at the same scale after sampling to overcome the problems of low dimensionality in the deep layer and the inadequate expression of small objects. The sizes of candidate regions are modified to fit the size of the actual airplanes in the remote sensing images. The fully connected layers are replaced with convolutional layers to reduce the network parameters and adapt to different input image sizes. The region proposal network shares convolutional layers with the detection network, which ensures high detection efficiency. The simulation results indicate that, when compared to typical airplane detection methods, the proposed method is more accurate and has a lower false alarm rate. Additionally, the detection speed is considerably faster and the method can accurately and rapidly complete airplane detection tasks in aerial remote sensing images.

## 1. Introduction

Airplane detection is used in many fields such as image reconnaissance, status monitoring, remote sensing analysis, and in other industrial or civil fields. It is relatively easy to detect airplanes in the air due to the simple sky background. However, images of airplanes on the ground contain interference from the surrounding terrain and are often small with multiple scales and color features. In these situations, airplane detection is extremely difficult.

An airplane detection method typically consists of two steps: locating candidate regions and classifying objects in the candidate regions. Different location and identification methods and various combinations of these methods can produce different detection results. Yildiz et al. [1] combined a Gabor filter with a support vector machine (SVM) for airplane detection. Liu et al. [2] proposed an airplane recognition method based on coarse-to-fine edge detection. Li et al. [3] used visual saliency and a symmetry detection method for airplane detection. Tan et al. [4] used a directional local gradient distribution detector to obtain a gradient textural saliency map and detected objects by segmenting the saliency map using a Constant False Alarm Rate (CFAR)-type algorithm. Wang et al. [5] proposed a novel method in two steps to overcome the problem of low aircraft detection precision in remote sensing images. First, an improved region-scalable fitting energy (RSF) algorithm was used to address the region of interest (ROI) extraction difficulties encountered with the presence of a complex background. Then, a corner-convex-hull-based segmentation algorithm was used to solve the aircraft shape irregularity problems. The above methods all use low-level features, such as edges and symmetry for detection. Therefore, they have a high false alarm rate and low calculation efficiency. As such, these methods cannot meet the requirements for rapid and accurate detection. Deep learning methods have been developed that can automatically learn object features. These methods extract sparse high-level features with strong representation ability. Consequently, the recognition ability has increased considerably. In one study [6], the binarized normed gradients (BING) method, which involves region proposals, was combined with a convolutional neural network (CNN) to perform airplane detection. This approach improved the detection performance compared to traditional methods and reduced the detection time. However, during the region proposal process, every image produced approximately 2000–3000 candidate regions, which were included in the same detection network, resulting in redundant calculations and decreased detection speed. A deep belief network was combined with an object positioning method to improve detection performance [7], but the positioning method was based on a sliding window search that consumed enormous amounts of time. Therefore, the method was slow. Zhang et al. [8] proposed a weakly supervised learning boxwork based on coupled CNNs. They mined and augmented the training data set by an iterative weakly supervised learning boxwork. They then combined a candidate region proposal network and a localization network to extract the proposals and simultaneously locate the aircraft. Zhong et al. [9] proposed a model that achieved favorable detection accuracy, especially for partially-occluded objects. However, this method requires optical images so is not suitable for remote sensing images. Li et al. [10] proposed an aircraft detection boxwork based on reinforcement learning and CNNs in remote sensing images. The limitation of their method was the large running time requirement when compared with state-of-the-art methods.

For airplane detection, training a rotational invariant classifier is necessary. To deal with aspect of airplane detection, Zhang et al. [11] used extending histogram-oriented gradients to obtain rotationally-invariant features. Alternatively, Wang et al. [12] proposed a rotation-invariant matrix to achieve the same. Although the above algorithms obtain the desired rotation invariance of the airplane, they are not easily scalable to other objects. In this paper, airplanes are generated at arbitrary orientations via data augmentation (rotation, flipping) and then a CNN is used to learn the rotational invariance of features directly, which is similar to learning the other features of the airplane.

To improve detection efficiency, Girshick et al. [13] proposed a detection network based on a spatial pyramid pooling method; the detection efficiency increased by several hundred-fold. To reduce the time required for candidate region generation, a region proposal network (RPN) was proposed [14]. In this case, the candidate region was generated by sharing features that were extracted from a detection network convolutional layer, and it required almost no extra time to generate candidate regions. Since multiple convolutional and pooling layers result in low dimensionality in the final feature map, a 32 × 32 object will be only 2 × 2 when it reaches the last convolutional layer of the Visual Geometry Group network (VGGNet) [13]. The feature map size is too coarse for classifying some instances that are small. At the same time, neighboring regions may significantly overlap each other. This is the reason why the detection results for small objects were not ideal. Long et al. [15] proposed a convolutional network that fused feature maps of the last few layers of the network. This process enriched the feature information and yielded a pixel-by-pixel semantic segmentation output. Xu et al. [16] proposed an end-to-end training method for a fully convolutional network to detect airplane objects. The authors obtained a high detection rate and reduced the detection time.

Inspired by the above concepts, this paper proposes an airplane detection method that fuses the multilayer features of a CNN. Using an RPN and an adaptive pooling network as a basic boxwork, regions are generated and identified using down-sampling and up-sampling methods to fuse the feature maps of different layers and thereby replace the final feature map. Specifically, the fully connected layers are replaced by convolutional layers, and the training and testing processes are accelerated using a Graphics Processing Unit (GPU), which increases the detection speed.

This paper provides the following contributions. (1) This research overcomes the problems of low dimensionality in the deep layer and the inadequate expression of small objects by fusing the shallow layer and deep layer features of a CNN on the same scale after sampling. (2) This research replaces the fully connected layers with convolutional layers to reduce the network parameters and to adapt to different input image sizes. (3) This research ensures high efficiency by sharing the convolutional layers of the region proposal network and the detection network. (4) The proposed method is an end-to-end approach used for effective, highly efficient, and real-time airplane detection.

## 2. Multilayer Feature Fusion

### 2.1. Classical Object Detection Network and Limitations

A classical object detection network is typically divided into two parts: region extraction and object identification [17]. Region proposal networks (RPNs) [14] can share convolutional layers with the object detection network, which can increase the efficiency of the extraction process. However, experiments have revealed that the results are often poor for small object detection, and positioning inaccuracy can be an issue. A detailed analysis of this issue is provided below.

#### 2.1.1. Network Structure and Characteristics

**Figure 1:**
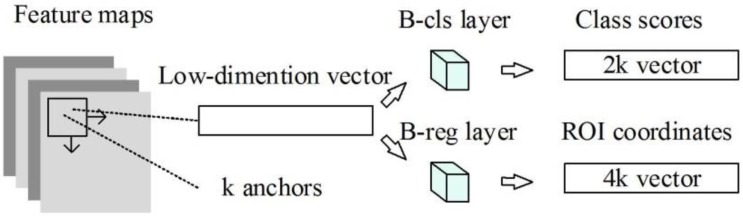
Schematic diagram of the region proposal network (RPN) structure. ROI = region of interest.

An RPN takes an image as input and outputs candidate regions. To extract candidate regions, a sliding window (typically 3 × 3) is slid over the feature map output by the last shared convolutional layer. Each sliding window position produces a low-dimensional vector from which candidate regions with different sizes and aspect ratios can be generated. Afterward, the vector feature is fed into two fully-connected sibling layers. These are the box-classification layer (b-cls layer) and the box-regression layer (b-reg layer). The b-cls layer estimates whether the candidate regions contain the objects and the b-reg layer adjusts location information. The number of candidate regions for each location is denoted as *k*. Therefore, the b-cls layer outputs 2*k* scores and the b-reg layer has 4*k* outputs encoding the coordinates of *k* locations. The network structure is shown in Figure 1.

**Figure 2:**
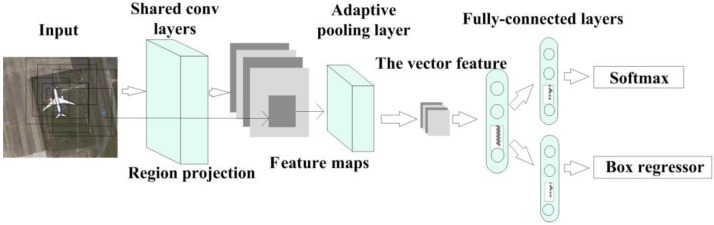
Diagram of the detection network.

The detection network we use in this study was previously proposed [18]. Based on spatial pyramid pooling, feature maps with different sizes are used to generate the output for a fixed-size feature map. This is appropriate since the input maps of the candidate regions are of different sizes. To reduce the calculation redundancy, the spatial location of the candidate region is matched with the corresponding feature map from the same input image, so the same network is not used repeatedly. Other structural parameters refer to the RPN, except for the classification layer, which is based on multi-object classification. The network structure is shown in Figure 2.

#### 2.1.2. Network Limitations

As the convolutional layers deepen, the feature map’s dimensionality continually decreases, the features become more abstract, and the semantic features become increasingly clear [19]. This means that position information becomes increasingly distorted, and position inaccuracy issues inevitably exist. A bounding box regression layer [20] can alleviate this problem to a certain degree, but a difference still exists among different input images in which the correction result may be poor. Multiple convolutional and pooling operations run alternately. Therefore, the feature maps corresponding to small objects are sparse and characterization can be inadequate, which can lead to poor detection performance for small objects.

### 2.2. Shallow and Deep Layer Feature Fusion for Convolutional Neural Networks

Two requirements can be summarized from the above section. First, the feature maps used for region proposal and detection should contain abundant information, including both semantic and position information. Second, the feature map should be of suitable size; a too-small map leads to inadequate feature representation, and being too large affects the calculation efficiency.

The features from deep layers have a high abstraction ability and abundant semantic information but lack position information. By contrast, the features from shallow layers are associated with precise positioning information for objects, but the semantic features are not clear and feature representation ability is poor [21]. Therefore, a good detection network should consider fusing the shallow and deep features with a specific intent to balance the semantic and positional information and obtain a feature map with a suitable size. In a previous study [22], multilayer feature maps were fused using the pooling, deconvolution, and weighted summation methods, which eventually led to a more accurate detection network.

**Figure 3:**
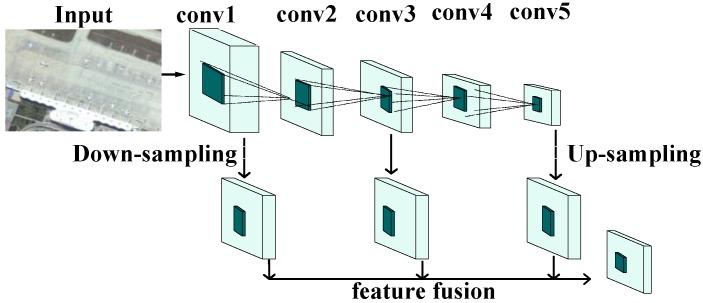
Structural diagram of a convolutional neural network (CNN) with multilayer fusion.

Based on this concept, in our work, the features from different layers were converted to the same scale using different sampling methods. Down-sampling was used for shallow-layer features, up-sampling was used for deep-layer features, and the middle-layer features were left unchanged. These features were processed in a convolutional layer before they were fused to generate a feature map with a suitable size. The generated feature map was used to replace the output feature map of the final convolutional layer, and furthermore was used to extract and identify candidate regions. The specific network structure is shown in Figure 3.

## 3. Methodology

### 3.1. Overall Boxwork and Technical Details of the Detection Network

The entire network structure is founded on a classical region-based convolutional neural network. The details that were modified to apply the network to airplane detection are described below.

(1) Since the size of an airplane in an image is usually small and its shape is mostly square, three relatively small sizes and three aspect ratios were selected for a total of nine candidate region generation parameter sets. The choices are summarized in Table 1.
(2) To handle input images with different sizes, the scale of the network was reduced. All fully connected layers were replaced by the corresponding convolutional layers. To fuse multi-level feature maps at the same resolution, different sampling methods were used for different layers. We added a maximum pooling layer on the lower layer to perform down-sampling. For higher layers, we added a deconvolutional operation to conduct up-sampling. The feature maps of different layers were subjected to a convolutional layer integral before fusion. Then, the feature maps were adjusted to the same size and the semantic features were simultaneously enhanced.
(3) Multilayer features fusion decreases the speed of the entire detection system. To increase the speed, a convolutional layer was inserted into the multilayer fusion feature map before generating a candidate region to reduce the dimensionality of the feature maps. This process reduced the time needed to generate the regions. However, the direct use of a reduced-dimensionality feature map for detection leads to a slight decrease in accuracy. Therefore, the feature map that was used during the detection process should be used before the convolution operation.
(4) In the training stage, an RPN generates tens of thousands of candidate regions, of which many are redundant or similar. To increase the candidate region quality, a non-maximal inhibition method [23] was used to filter the candidate region set. Approximately 1000 high-quality candidate regions are kept, and the first 200–300 regions were used as a training set. For the positioning accuracy of the bounding box, we used the intersection-over-union (IoU) formula. The IoU indicates the degree of overlap between bounding box *A* and the ground truth *B*, as shown in Equation (1):
  (1) IoU=(A∩B)/(A∪B)

A binary label was assigned to a candidate region (whether an airplane exists) as a training sample. The two conditions for a positive sample were: (1) an IoU that is greater than 0.75 for any manual calibration boundary box and (2) a maximum IoU that could be less than 0.75 for a certain manual calibration boundary box. One manual calibration boundary box could have many corresponding positive samples. The condition for a negative sample was that the IoU was lower than 0.3 for all manual calibration boundary boxes. Other samples did not affect the training. To ensure a balance between the positive and negative samples in the training process, 100 positive and negative samples were randomly generated to form a small batch.

(5) A previous study indicated that multitask loss joint training can result in supplementing information among tasks, which improves common feature extraction. Therefore, the multi-task loss in the literature [13] was used to jointly train the classification and boundary box regression tasks. We define the loss function as:
  (2) L({pi},{ti})=1Ncls∑iLcls(pi,pi∗)+λNreg∑ipi∗Lreg(ti,ti∗)
  where *i* is the index of a candidate region during training and *p_i_* is the probability that region *i* contains an object. If the sample is positive, then p_i_* = 1, and for a negative sample, p_i_* = 0. *t_i_* is a vector representing the four parameterized coordinates of the predicted boundary box and t_i_* is associated the ground-truth box, which is associated with a positive sample. The outputs of the classification layer and boundary box regression layer are {*p_i_*} and {*t_i_*}, respectively. *N_cls_* and *N_reg_* are normalization factors. Typically, *N_cls_* is the number of small batch samples (i.e., *N_cls_* = 256) and *N_reg_* is the number of candidate regions (i.e., *N_reg_* ≈ 2400). To roughly equally weight the two terms in Equation (2) after normalization, we set *λ* = 10.

The classification loss *L_cls_* and bounding box regression loss *L_reg_* are expressed in Equations (3) and (4), respectively, where *u* is the classification type index.

(3) $$L_{cls}(p,u)=-\log p_{u}$$

(4) $$L_{reg}(t_{i},t_{i}^{*})=R(t_{i}-t_{i}^{*})$$

In these equations, *R* is determined from the literature [6] as:

(5) $$R(x)=\left\{ \begin{array}{c} 0.5x^{2}\;if\left|x\right|<1\\ \left|x\right|-0.5\;else \end{array}\right.$$

(6) The training of the detection network is based on the region extracted by the RPN. Therefore, these two networks cannot be simultaneously trained. Instead, the training occurs in sequence. For multiplex use of a feature extracted from a convolutional layer by the two networks, the cross-optimized training strategy proposed in a previous study [14] was used. The training steps are as follows.

Step 1: Select the pretrained convolutional network and discard the layers behind the final convolutional layer. The initial values from these networks are used for the region proposal and detection networks and a complete network model is constructed according to the network structure.

Step 2: Train the region proposal task of the network and fine tune the network weights. Use the feature maps obtained after the multilayer feature fusion to generate a candidate region and discard the redundant regions using a non-maximum inhibition operation. Finally, select the 200 regions with the highest score from the input image classification as the training sample.

Step 3: Use the training sample produced in the second step to train the detection network and fine tune the network weights. Note that the detection network initialization method is the same as in the first step (i.e., not based on the fine-tuned network weights in the second step).

Step 4: Use the convolutional layer weight of the detection network trained in the third step as an initialized convolutional layer and keep the layer fixed. Train the region proposal network task again and generate new candidate regions. Use the same method used in the second step to select the candidate regions for the training sample.

Step 5: Keep the convolutional layer in the fourth step fixed and use the newly generated training sample to train the detection network. At this point, the convolutional layer of the two networks is shared. The training of the entire network is complete, and the network can be used for airplane detection.

### 3.2. Network Model Selection and Initialisation

Training a new mission-specific network requires datasets with a large number of labelled images. However, constructing such labelled datasets requires an enormous amount of manual work. Additionally, the more annotations the dataset requires, the easier it is for omissions and errors to occur. Research revealed that the low-level features of convolution networks extracted from different types of image objects are similar. Additionally, the weight value of network training based on a general dataset can be applied for specific detection tasks. This approach is the more popular transfer learning method [24]. Some researchers applied transfer learning for airport detection [25] and obtained sufficient results. This paper uses a network that was pretrained on the large ImageNet dataset [26] to acquire the initial weight values for the model. Then, the network weights were fine-tuned by retraining with a smaller, annotated dataset. To avoid overfitting for airplane detection, a small network pre-trained with Zeiler and Fergus nets (ZF nets) [27] was selected as the initial value of the convolution layer, and the other network structures used a 0 mean and 0.0001 variance Gaussian function for random initialization.

## 4. Simulation Experiment and Analysis

### 4.1. Simulation Platform

We used MATLAB 2014b as the simulation platform and Visual Studio 2013-compiled Caffe as the neural network boxwork. The computer configuration was an I7-7700 3.6 GHz CPU with 16 GB of memory and an NVIDIA GTX 1060 GPU.

### 4.2. Data

All airplane images were collected from satellite images of the world’s 200 airports in Google Earth, including Ronald Reagan Washington National Airport (N 38.85°, W 77.04°), Beijing Capital International Airport (N 40.07°, E 116.59°), etc. The images were collected during December 2017. The resolutions of the images ranged from 1 to 10 m. The image sizes were between 800 × 600 and 1200 × 800. Due to our research needs, the database is temporarily unavailable.

**Figure 4:**
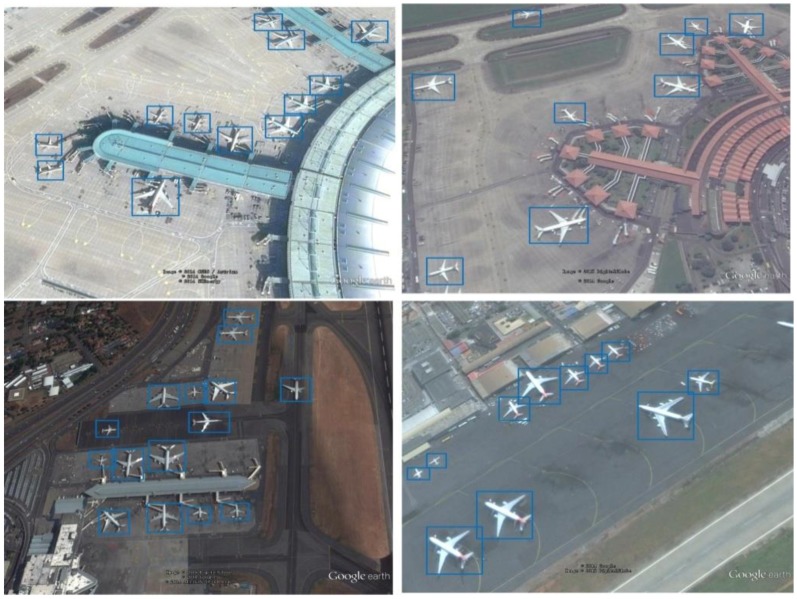
Examples of training data.

Additionally, 50% of these images were randomly selected as training and validation data and the remaining images created the test dataset. To overcome the airplane rotation issue and prevent overfitting, the training images were rotated by 90°, 180°, and 270° and horizontally flipped for data augmentation. With these modifications, there were a total of 250 training data points. We completed the data labeling work for training and testing. Some examples of the training data are shown in Figure 4. In this paper, the only object we wanted to detect was an airplane, so we only labeled the airplane’s location during annotation. As shown in Figure 4, the blue rectangles are the ground-truth boxes. The annotated data were input directly to the CNNs for training to realize end-to-end training.

Airplane detection in this paper focuses only on whether the image contains an airplane, which is a binary classification problem. The region occupied by airplanes in an image is small, and one image contains approximately 10–20 airplanes. The training dataset contained approximately 4000 airplanes, which was sufficient to train a ZF Net and avoid overfitting.

### 4.3. Simulation Experiment with Training and Testing

**Figure 5:**
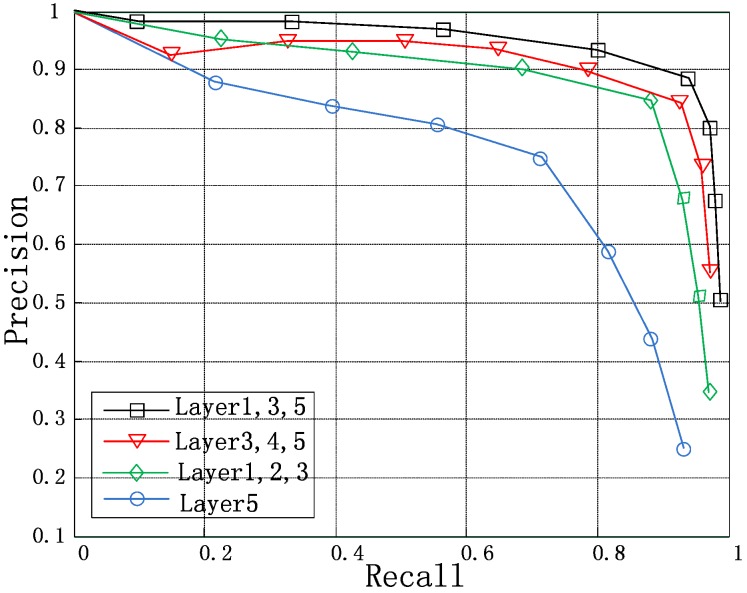
Precision-recall curves of the network obtained by fusing the features of different layers.

To explore the influence of fusing the feature maps of different layers on the detection result, we used the same training method to perform multiple sets of comparison experiments. We fused layers 1, 2, and 3 in one experiment; layers 3, 4, and 5 in another; and layers 1, 3, and 5 in a final experiment. To ensure the selection of the first 300 extracted regions from each training image, we calculated the accuracy and recall rate. The experiment results are shown in Table 2. To intuitively demonstrate the performance of different fusion methods, precision-recall (P-R) curves are shown in Figure 5.

The results in Table 2 and Figure 5 show that fusing the features of different layers can significantly influence the detection results. When using only the fifth layer and not fusing the other features, the network airplane detection performance is poor because the airplane objects are extremely small and the occupied region in the input image is too small. Therefore, the feature map obtained through multiple convolutions and poolings is small and lacks characterization ability. The detection performance of the network for airplane detection significantly increased after the fusion of the multilayer features, which indicates that this type of fusion can enhance the features characterization ability. The fusing different sets of layers, such as layers 1, 2, and 3; layers 3, 4, and 5; and layers 1, 3, and 5, did not produce a significant difference, but the fusion of layers 1, 3, and 5 performed the best. Neighboring layers exhibited a high correlation, which insufficiently concentrated the fused features. Nonetheless, fusing multilayer features had a positive effect on the detection results.

**Figure 6:**
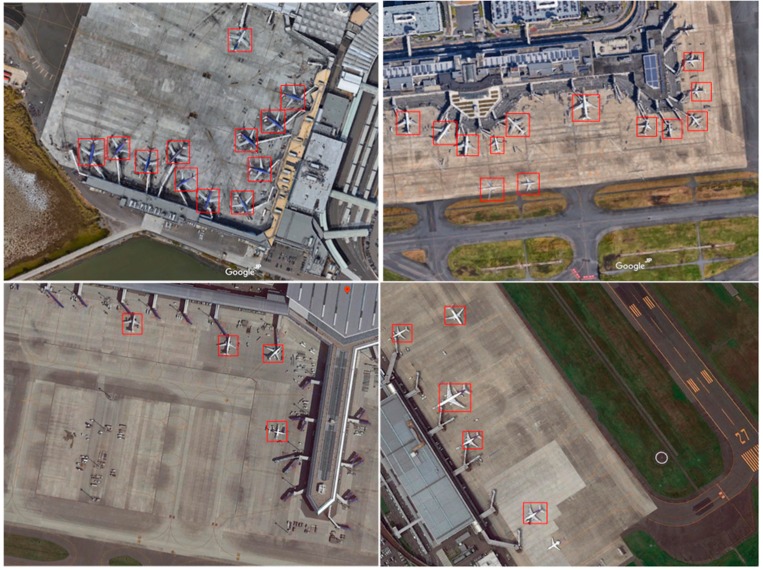
Results of the proposed airplane detection method.

Figure 6 shows the detection results for four images. All of these images were obtained using the network based on the fusion of layers 1, 3, and 5. The figure shows that the proposed method yields very good detection results for small objects such as airplanes.

**Figure 7:**
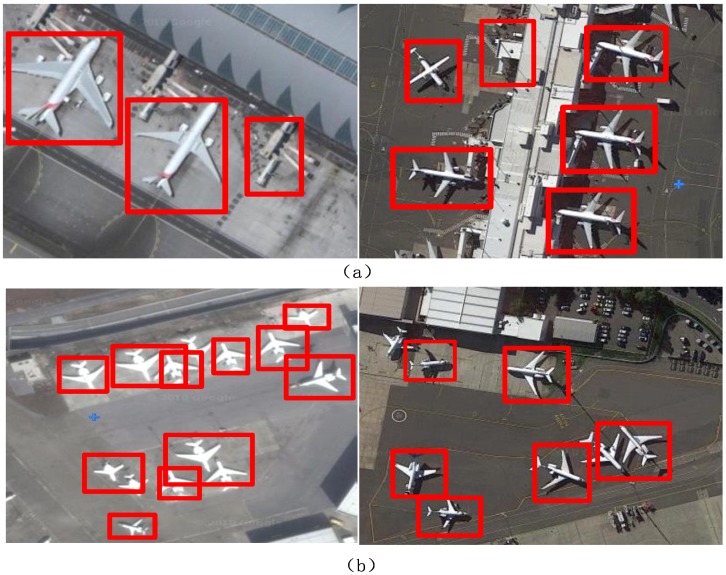
Various airplane detection failures. (**a**) Some regions were misidentified as airplanes; (**b**) many airplanes were not determined to be airplanes.

Some airplane detection failures are shown in Figure 7. In Figure 7a, the method misidentified the region as an airplane because the object is similar to an airplane. In Figure 7b, the airplanes are cluttered and their rectangular windows overlap areas are larger. Therefore, many airplanes were not determined to be airplanes. This method greatly improved the ability to detect small objects. Although the above two situations are not very likely to occur, the method still needs improvement. The direction of our next research study will focus on using contextual information.

### 4.4. Comparison with Other Methods

To examine the performance of the proposed method, another 125 airplane images were selected as the testing dataset. Two types of typical airplane detection methods were compared. Three indicators were analysed: the final detection rate, false alarm rate, and average detection time. “Location-DBN” refers to the airplane detection method based on positioning and a deep confidence network from the literature [7], and “BING-CNN” refers to the airplane detection method based on the region extraction method “BING” and a convolutional neural network [6]. Fast RCNN refers to a method that was modified from a previous approach [13], and a new model was developed by using the airplane dataset to retrain the detection portion of the network. Faster RCNN refers to a method based on the literature [14] and was modified to vary the size of the candidate boundary box of the region extraction network. A new model was then obtained by using the airplane dataset for re-training. In order to avoid errors, the results of each method were averaged based on three experimental trials. The platform, data, and method of each experiment were consistent. For comparison, all values retain a single digit after the decimal point. The results are shown in Table 3.

In this table, the detection rate and the false alarm rate were calculated using:

(6) $$\mathrm{Detection}\;\mathrm{rate}=\frac{\mathrm{Number}\;\mathrm{of}\;\mathrm{correctly}\;\mathrm{detected}\;\mathrm{airplane}}{\mathrm{Number}\;\mathrm{of}\;\mathrm{all}\;\mathrm{airplanes}}\times 100\%$$

(7) $$\mathrm{False}\;\mathrm{alarm}\;\mathrm{rate}=\frac{\mathrm{Number}\;\mathrm{of}\;\mathrm{incorrectly}\;\mathrm{detected}\;\mathrm{airplane}}{\mathrm{Number}\;\mathrm{of}\;\mathrm{all}\;\mathrm{detected}\;\mathrm{airplane}}\times 100\%$$

In the above comparative experiments, the proposed method fused layers 1, 3, and 5, and the first 300 candidate regions were selected for the region extraction stage. Notably, the latter three methods all used GPU acceleration. Without this advantage, the detection time would increase more than 10-fold. Under this condition, the latter three methods in the table did not exhibit a significant speed advantage, but the latter two methods exhibited better overall performance than the first two methods. Table 3 shows that the proposed method exceeds the other three methods in terms of the detection rate, false alarm rate, and detection time. Although the detection time of the proposed method is slightly longer than that of the Faster RCNN method, the detection rate and the false alarm rate are much better. Overall, the comprehensive performance of the proposed method is better than that all the other methods. This difference is due to the powerful feature characterization capabilities of CNNs. Moreover, the feature fusion of the shallow and deep layers enhances the ability of the network to detect small objects.

## 5. Conclusions

This paper proposed an airplane detection method that fuses features from different layers on the same scale after sampling. The positional information of the shallow layers was fused with the semantic information of the deep layers to improve the ability of feature characterization. Additionally, this approach addressed the problems of the low dimensionality of the deep features and the low capability for small object expression. Candidate boxes of nine sizes were used to consider different scales of airplanes in the images. The convolutional layers replaced the fully connected layers to reduce the network parameters, which was appropriate for the different sized input images. The region proposal network shares convolutional layers with the detection network, which ensures high detection efficiency. The proposed method was used for effective, highly efficient, and real-time airplane detection and can be applied to real-time detection of different types of objects.

Despite the superior performance, our method still has some limitations. As described in Section 4.3, some false positives and missed detections occurred. Our method needs improvement. It is well known in the study of computer vision that context plays an important role in visual recognition. Thus, our future research will focus on the use of contextual information.

## Figures and Tables

**Table 1:** Boundary box dimensions for nine candidate regions.

**Set**	30^2^, 1:1	60^2^, 1:1	110^2^, 1:1	30^2^, 4:5	60^2^, 4:5	110^2^, 4:5	30^2^, 5:4	60^2^, 5:4	110^2^, 5:4
**Size**	30 × 30	60 × 60	110 × 110	27 × 33	53 × 67	98 × 122	33 × 27	67 × 53	122 × 98

**Table 2:** Simulation experiment results from fusing the features of different layers.

Layer(s)	5	1 + 2 + 3	1 + 3 + 5	3 + 4 + 5
Precision	79.7%	89.3%	95.5%	92.1%
Recall	75.4%	86.4%	90.1%	88.6%

**Table 3:** Comparison of the proposed method with two comparative methods.

Method	Location-DBN	BING-CNN	Fast RCNN	Faster RCNN	Our Method
Detection rate (%)	83.5	85.4	81.2	82.7	95.5
False alarm rate (%) Average time (s)	36.5 >100	20.4 6.1	22.5 2.9	23.5 0.2	7.5 0.3
